# Effects of Chromium Exposure on the Gene Expression of the Midgut in Silkworms, *Bombyx mori*

**DOI:** 10.3390/genes14081616

**Published:** 2023-08-12

**Authors:** Wantao Rong, Yazhen Chen, Jieyou Lu, Shuiwang Huang, Lei Xin, Delong Guan, Xiaodong Li

**Affiliations:** 1 Guangxi Key Laboratory of Sericulture Ecology and Applied Intelligent Technology, Hechi University, Hechi 546300, China; 18023@hcnu.edu.cn (W.R.);; 2 Guangxi Collaborative Innovation Center of Modern Sericulture and Silk, Hechi University, Hechi 546300, China; cyz2013060415@163.com (Y.C.);

**Keywords:** chromium exposure, silkworms, *Bombyx mori*, stress response genes, comparative transcriptomics

## Abstract

Chromium is a severe heavy metal pollutant with significant environmental risks. The effects of Chromium on the digestion of *Bombyx mori* (silkworms) are of particular importance due to their ecological and economic significance. Herein, RNA sequencing was conducted on nine midgut samples from silkworms exposed to control, 12 g/kg and 24 g/kg Chromium chemical diets. Comparative transcriptomics revealed that under moderate Chromium exposure, there was a significant increase in up-regulated genes (1268 up-regulated to 857 down-regulated), indicating a stimulation response. At higher stress levels, a weakened survival response was observed, with a decrease in up-regulated genes and an increase in down-regulated genes (374 up-regulated to 399 down-regulated). A notable shift in cellular responses under medium chromium exposure was exposed, signifying the activation of crucial metabolic and transport systems and an elevation in cellular stress and toxicity mechanisms. The observation of up-regulated gene expression within xenobiotic metabolism pathways suggests a heightened defense against Chromium-induced oxidative stress, which was primarily through the involvement of antioxidant enzymes. Conversely, high-dose Chromium exposure down-regulates the folate biosynthesis pathway, indicating biological toxicity. Two novel genes responsive to pressure were identified, which could facilitate future stress adaptation understanding. The findings provide insights into the molecular mechanisms underlying silkworms’ digestion response to Chromium exposure and could inform its biological toxicity.

## 1. Introduction

Chromium, a heavy metal and one of the significant elements in the earth’s crust, can contaminate soil and water, leading to bioaccumulation in the food chain and causing adverse effects on the health and survival of various species, including insects [1,2]. Consequently, Chromium pollution significantly threatens the environment and living organisms [3,4,5]. Extensive studies have shown that Chromium exposure can induce severe toxicity, such as reducing the egg-hatching rate in mayflies (*Ephemera orientalis*) [6], causing DNA damage and neurotoxicity in files (*Drosophila melanogaster*) [7,8], and inhibiting growth rate and reproduction in silkworms [9,10]. According to the review by DesMarias and Costa, Chromium can exist in different oxidation states, namely trivalent (Cr(III)) and hexavalent (Cr(VI)). Both trivalent (Cr(III)) and hexavalent (Cr(VI)) can induce cellular stress and lead to adverse health effects. Hexavalent Chromium (Cr(VI)) enters cells through general sulfate transporters on the cell surface and exerts its toxic effects following reduction by ascorbate and biological thiols such as glutathione (GSH) or cysteine. The removal of Cr(VI) by ascorbate generates Cr(IV) intermediates, while reduction by GSH produces reactive Cr(V), both of which can ultimately be converted to Cr(III). This reduction process can generate high levels of oxidative stress, leading to damage to cellular lipids, proteins, and DNA.

Additionally, reintroducing ascorbate into the cell culture medium decreases oxidative stress but induces DNA double-strand breaks by forming ternary DNA adducts containing Cr(III) cross-linked with various molecules [11]. Given the attention that Chromium exposure in insects has received, research focused on investigating the genetic changes required in critical genes is necessary [12]. However, such research remains rare in most insects, including model insect species such as flies and silkworms [13]. Understanding the molecular mechanisms underlying these effects is crucial for developing strategies to mitigate the impact of Chromium pollution on ecosystems and the biotoxicity of insects.

Silkworms (*Bombyx mori*) were selected as the research subject for this study based on two factors. Firstly, silkworms are economically significant insects due to their role in silk production, making their health and survival a matter of concern. Sericulture, the breeding of silkworms for silk production, often occurs in natural environments, exposing the silkworms to various familiar sources of Chromium exposure. The prevalence of chromium exposure and its potential toxicity in silkworms have been a subject of interest. In the environment, trivalent Chromium is naturally present in soil and can be found in foods and nutritional supplements. However, hexavalent Chromium is predominantly released into the atmosphere through human activities and can contaminate air and drinking water sources [11,12,14,15]. Therefore, finding breeds more resistant to heavy metal stress is a long-term goal of scientific breeding [16,17]. Secondly, the high-quality silkworm reference genome has already been sequenced, providing a solid foundation for studying gene expression changes under Chromium exposure [18,19,20]. Using reference genome-guided transcriptomic analysis, we can investigate the advantages of understanding gene expression changes in silkworms under Chromium exposure. Previous research utilizing referenced transcriptomics has demonstrated that heavy metal stress such as Cadmium [21], Lead [22], and Zinc [23] can alter gene expression, affecting growth, reproduction, and overall fitness through the scavenging of reactive oxygen species (ROS) [24,25,26]. Several essential genes encoding enzymes involved in these processes, such as superoxide dismutase (SOD), cytochrome P450, and glutathione S-transferase (GST), were also identified [27,28,29]. Similarly, we can now reveal the unique molecular mechanisms underlying the response to Chromium exposure by examining the impact of Chromium exposure on the silkworm transcriptome. Comparing these findings with previous studies on other heavy metals will also allow a better understanding of the similarities and differences in the molecular responses to various heavy metal stresses.

In this study, we comprehensively evaluate Chromium exposure’s transformative effects on the gene expression profile in silkworms. With the experimental design incorporating chromium supplementation in the silkworm diet, we simulated a stress scenario linked to heavy metal intake. Following dual-treatment regimens, high-resolution transcriptomic sequencing was deployed across control, medium, and high-concentration cohorts to capture shifts in gene expression in the midgut—the primary site of digestion. This holistic transcriptomic approach allowed for an intricate deciphering of the underpinning molecular mechanisms triggered by chromium exposure. Our inquiry explores chromium toxicity’s influence on gene expression in silkworms; it seeks to identify critical genes and pathways implicated in the silkworm’s response to heavy metal stress. The elucidated insights from this investigation promise to substantially contribute toward strategizing measures to mitigate the detrimental impact of chromium pollution on silkworms. This study further primes future research to expand our understanding of heavy metal-induced stress responses in insects.

## 2. Materials and Methods

### 2.1. Sample Collection

The silkworm samples used in this study were obtained from the Gui Cans 5th strain, a domestic representative silkworm species which had been reared in the laboratory of Hechi University for an extended period. Each group of silkworms was monitored for five days (120 h), which is a period corresponding to stage five of silkworm development. The control group was fed with non-Chromium-added feed, while the middle group was provided with feed containing 12 g/kg Chromium chloride (50% half-lethal dose), and the high group was fed with a meal containing 24 g/kg Chromium chloride (half-lethal dose) for 120 h (5 days, stage five). Following this period, 10 live individuals were randomly chosen from each experimental group and dissected on ice to obtain muscle tissue. The harvested tissue was stored in a −80 °C freezer to be used for subsequent RNA extraction and sequencing analysis. Eventually, nine samples were retrieved and grouped into three categories based on chromium exposure levels. Each sample consisted of pooled dissected muscle tissue from the previous ten individuals.

### 2.2. RNA-Extraction and Sequencing

The total RNA of the control and treatment groups were extracted from silkworm muscle tissue using the Qiagen RNA extraction kit (Qiagen, Valencia, CA, USA) according to the manufacturer’s instructions. RNA integrity was assessed using the Agilent 2100 Bioanalyzer (Agilent Technologies, Palo Alto, CA, USA) to ensure that RNA samples had an RNA integrity number (RIN) between 8 and 10. The qualified RNA samples were then subjected to cDNA library construction and sequencing using the Illumina HiSeq X-ten sequencing platform (Illumina, San Diego, CA, USA). A brief technical workflow for the cDNA library and sequencing procedure is described below: RNA was reverse transcribed to cDNA using a cDNA synthesis kit and library preparation using Illumina TruSeq Stranded mRNA Library Prep kits (Illumina, San Diego, CA, USA). The produced cDNA libraries were checked for quality and size distribution using an Agilent 2100 Bioanalyzer, and Qubit quantified their concentrations. Sequencing was performed using an Illumina HiSeq X-ten platform with an insert size of 380 bp and producing 150 bp paired-end reads. For each sample, at least 6 Gb of raw data was obtained. We entrusted Shanghai Yaoen Biotechnology Co., Ltd (Yaoen Biotechnology, Shanghai, China). to complete this study’s RNA extraction and sequencing work. The authors designed the experimental process and approved and reviewed the data produced by the company.

### 2.3. Genome-Guided Referenced Comparative Transcriptomic Analyses

Raw sequencing data were processed to generate raw fastq files through base calling. The quality control of raw data was performed using FastQC v0.12.1 [30] and Trimmomatic v0.38 [31] software to filter out low-quality reads (Q30 < 90%), ambiguous bases (N bases), trim adapters, and assess the quality of the reads. The genome-guided transcriptome analysis was performed using the hisat2 v2.2.1 [32] + stringtie v2.2.1 [33] + Deseq2 v3.42.4 [34] pipeline with the reference genome GCF_014905235.1_Bmori_2016v1.0_genomic.fna (https://www.ncbi.nlm.nih.gov/genome/?term=txid7091, accessed on 5 June 2023, 460.35 Mb) [18]. The clean reads for nine RNA-seq samples were grouped into three experimental groups: control (Treatment Control A group, TKA), middle concentration (Treatment 12 g/kg medium B group, T2B), and high concentration (Treatment 24 g/kg high C group, T4C). Firstly, the pre-processed reads were aligned with the reference genome using Hisat2 v2.21 software. Then, the aligned bam file was then subjected to transcript assembly using StringTie v.2.21 software to generate transcript expression levels and convert them into gene expression levels. The FPKM values were calculated at this step to quantify gene expressions. Afterwards, the DESeq2 R package v3.42.4 performed differential gene expression analysis between the experimental groups. The screening criteria were set as follows: *p*-value < 0.05 and log2 (fold change) > 1. The final significant DEGs from the T2B and T4C groups were provided in Supplement Tables S1 and S2. Heatmaps and volcano plots were generated using R software to visualize the differentially expressed genes among the three experimental groups.

### 2.4. Custom Data Analyses and Illustration

The functional annotation of the gene is inherited from the reference genome and is implemented by a shell script written by ourselves (Supplement Table S3). The differentially expressed genes were subjected to functional annotation and pathway enrichment analysis using the G.O. (gene ontology) [35] and the KEGG database [36]. The detailed enrichment for G.O. and KEGG are shown in Supplement Tables S4–S7. R packages, including the corrplot, factoextra, and ggplot2, were employed to conduct correlation and PCA analyses and draw customized figures, respectively.

All raw data were uploaded to the Genome Sequence Archive database of the China National Center for Bioinformation website under the Bioproject accession of PRJCA018238.

## 3. Results

### 3.1. RNA-seq Data Statistics and Quality Check

Overall, we successfully obtained data from nine RNA sequencing samples. The average number of clean reads per sample exceeded 40 million, with an average total sequencing amount of over 6 Gb (Table 1). In an eloquent parade of data evidenced by DNA sequencing, a relatively minor degree of variance interplayed among the samples, bringing forth intriguing results. Strikingly, the group assailed with a medium chromium concentration emerged as the outlier with the least sequencing yield, circling a modest 5.26 gigabases (Gb). The subsequent alignment to the reference genome also showed promising results, with all samples having a mapping rate above 85% and exceeding 95% in the T4C group. This high mapping rate meets the quality requirements for transcriptome analysis of the same species in different physiological states (generally, the mapping rate is over 75% according to our experience). In summary, the sufficient sequencing amount and good alignment results confirm the reliability of our data and provide solid support for our subsequent comparative transcriptome analysis.

**Figure 1:**
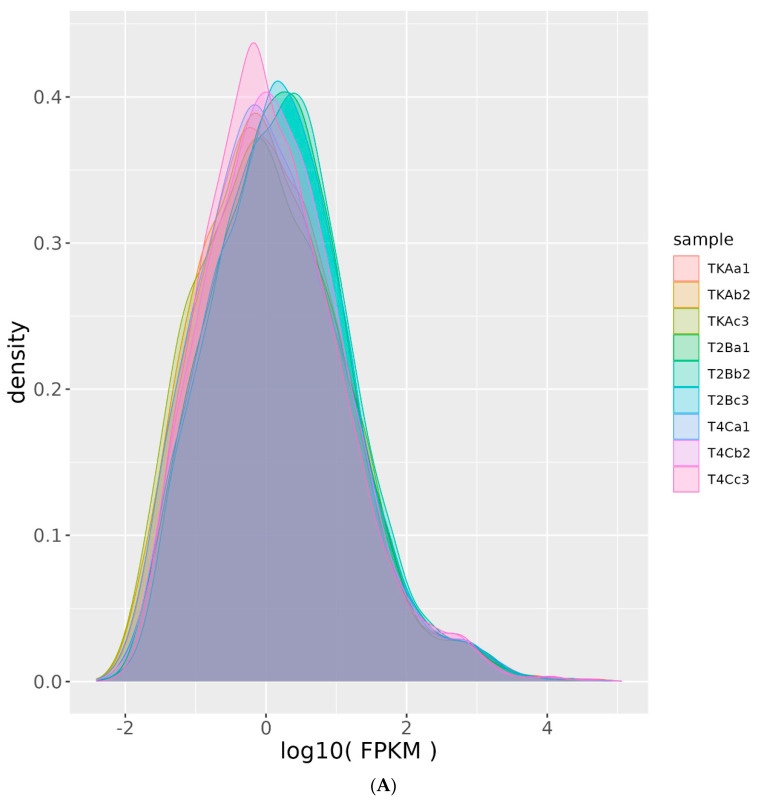

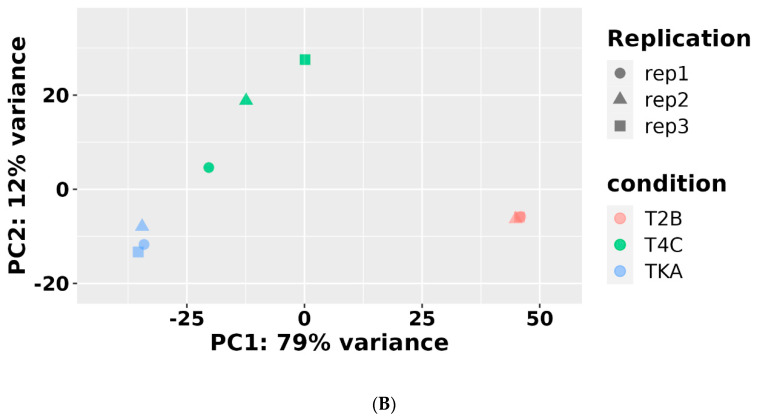
The FPKM density distribution (**A**) and PCA distribution (**B**). The FPKM density distribution graph (**A**) reflects changes in gene expression intensity across the samples measured. A peak in this distribution shows the most frequent expression level across the analyzed dataset. The PCA distribution chart (**B**) visualized the variation within the dataset. The distributions of dots positively represent the relationship of the samples, indicating a certain level of similarity. The PC1 and PC2 axes represent the first two principal components, respectively, which account for the major proportion of data variability.

Subsequently, we further evaluated the reliability of gene expression in our study data using FPKM density distribution and principal component analysis (PCA) (Figure 1A,B). The results showed that the FPKM distribution of each sample almost completely overlapped, indicating no significant differences due to factors such as sequencing amount or sequencing gene preference (Figure 1A). This demonstrated that the sample FPKM differences reflect gene expression differences rather than systematic errors. In the PCA analysis, the first and second axes explained over 90% of the total variation, indicating that each sample’s overall FPKM values are close in range and dimension. Each instance was clearly distinguished by its treatment group, showing good consistency in gene expression within each group and the ability to reveal differences in gene expression patterns between groups (Figure 1B). Each group has a distinguished FPKM usage pattern, indicating wide gene alternative expressions.

### 3.2. Differentially Expressed Genes

**Figure 2:**
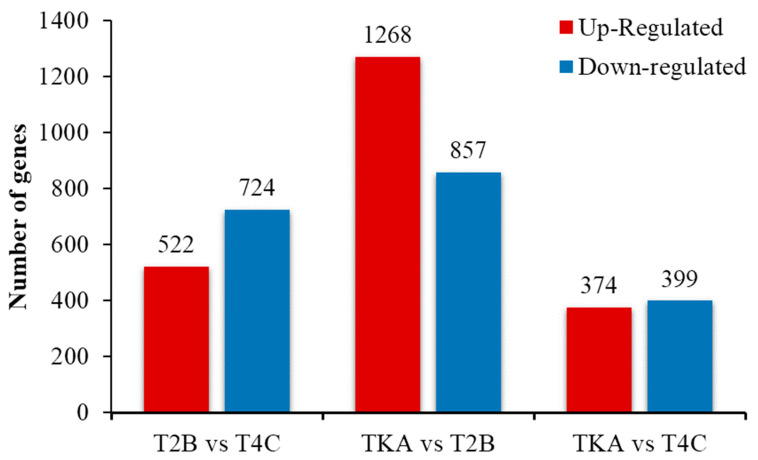
The histogram shows the number of up and down-regulated genes through comparisons among different groups.

Taking the control group (TKA) as a reference, we compared the changes in gene expression levels between the medium (T2B) and high (T4C) concentration treatment groups and attempted to identify patterns. Our findings showed that under medium concentration Chromium exposure, there were 1268 up-regulated genes and 857 down-regulated genes, with more up-regulated genes indicating a characteristic of stimulation response. Conversely, under high-concentration Chromium exposure, the number of up-regulated and down-regulated genes were 374 and 399, respectively, with fewer up-regulated genes than down-regulated ones (Figure 2). Notably, a lower count of differentially expressed genes was discerned under medium-concentration stress, pointing toward diminished survival traits under substantial toxicity. Observing this expression progression hinted at an adaptive response model that the silkworm might adopt under escalating levels of Chromium exposure, which was observable specifically at lower doses. However, a toxicity-mediated condition, chiefly driven by harmful effects, conceivably emerges in the intermediary space between the half-lethal and the 50% lethal concentration. To further investigate this supposition, we subjected the high-concentration cohort to a comparative study with the medium-concentration group. Remarkably, this comparison presented a parallelism in the figures of down-regulated and up-regulated genes across the two stress-level strata (Figure 2).

**Figure 3:**
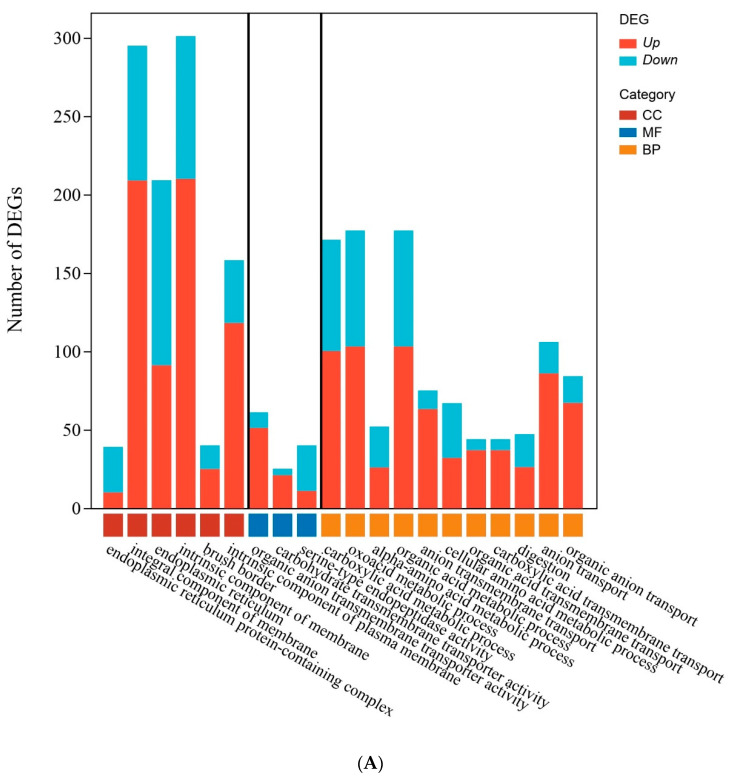

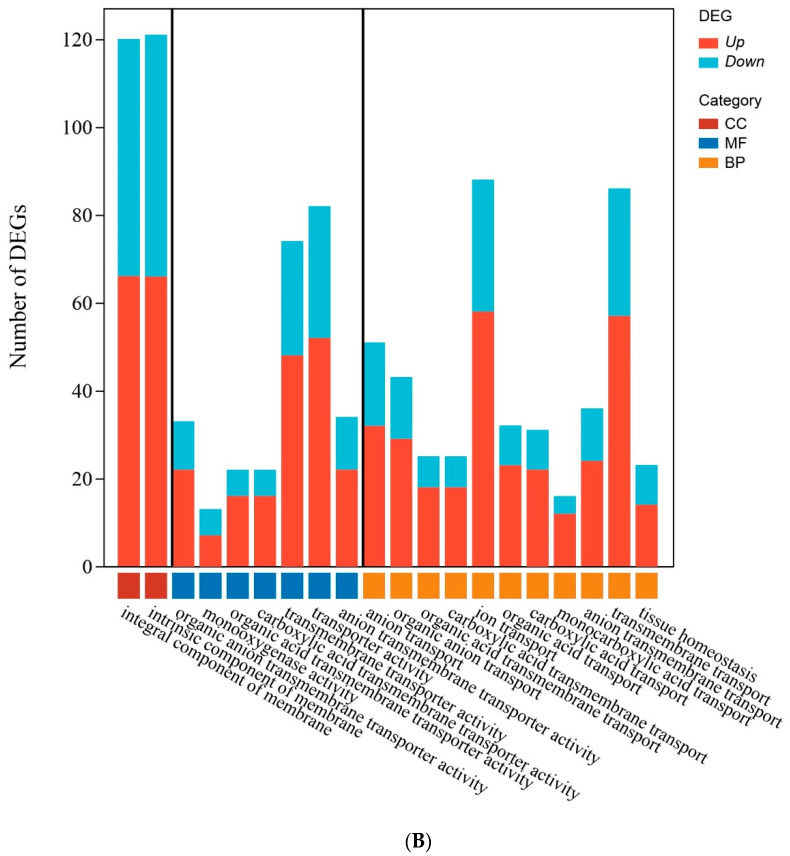
G.O. enrichment terms for all regulated DEGs in the medium-concentration treatment group (**A**) and high-concentration treatment group (**B**).

Subsequently, the attributes and functional implications of differentially expressed genes (DEGs) were described to understand the biological effects. G.O. enrichment analysis on medium- (T2B) and high-concentration (T4C) treatment groups compared to the control group (Figure 3A,B) showed that DEGs in the T2B group were notably enriched in molecular function and biological processes related to transport and transmembrane activity (Figure 3A). This suggests that transport and transmembrane-related enzymes are essential in helping cells transfer or segregate heavy metal ions during the silkworm’s response to Chromium exposure. The T4C group showed similar enriched gene functions, with membrane transport still dominant but with a unique monooxygenase activity pathway and biological processes involved in stress response and adaptation mechanisms (Figure 3B). The different activation of the pathways between the T2B and T4C groups indicate that in silkworms, the stress response mechanism may depend on the dose of Chromium.

### 3.3. Implications of the Underlying Biological Mechanisms

**Figure 4:**
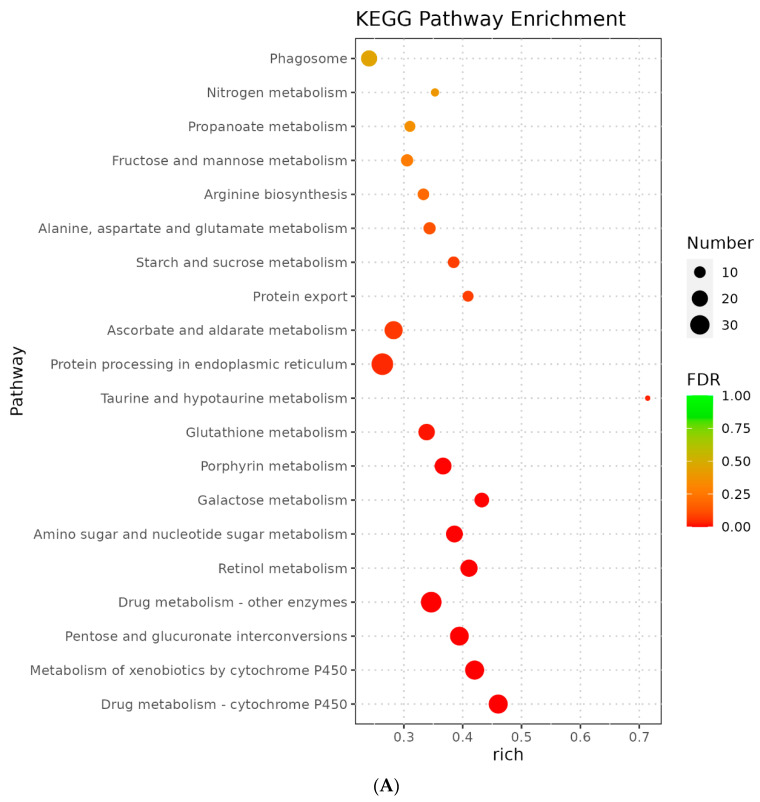

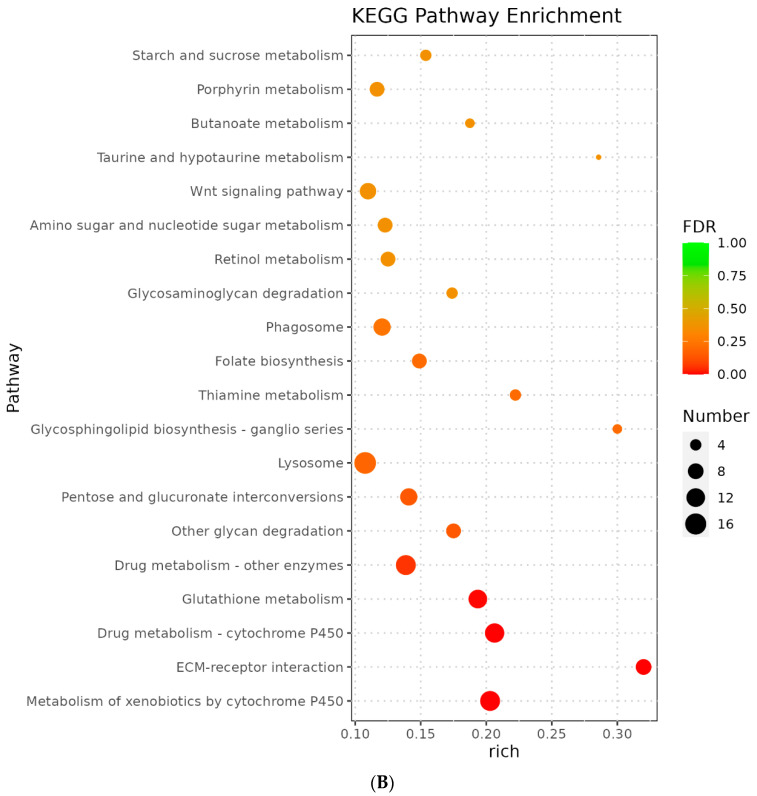
KEGG enrichment terms for all DEGs in the medium concentration treatment group (**A**) and high concentration treatment group (**B**). The horizontal axis represents the enrichment parameter of the pathway, while the size of the dots denotes the number of enriched genes—the larger the dot, the larger the quantity. The dot color corresponds to the FDR value; a lower FDR value leans more toward red, indicating a more significant enrichment of the pathway.

Furthermore, to gain a general comprehensive insight into the effects of Chromium treatment on silkworm gene expression profiles, we conducted enrichment analyses of differentially expressed genes (DEG) identified in both the moderate (T2B) and high (T4C) concentration groups using KEGG pathways in insects. Our results demonstrated that the T2B group had significant enrichment in KEGG pathways such as drug metabolism, carbohydrate metabolism, post-transcriptional modifications, degradation of antioxidants, and cell membrane transport (Figure 4A). Several *cytochrome P450* genes were identified in the drug metabolism pathway, indicating their potential role in metabolizing and degrading toxic chemicals to protect cells. Carbohydrate metabolism pathway enrichment suggested interference in energy metabolism, while the degradation of antioxidants and cell membrane transport processes directly related to oxidative stress may degrade ROS and transport metal ions to the extracellular space.

In contrast, the KEGG enrichment analysis of differentially expressed genes in the T4C group mainly included pathways related to metabolism, drug metabolism, and signal transduction (Figure 4B). These pathways comprised crucial developmental terms such as glycosphingolipid biosynthesis, folate biosynthesis, glycosaminoglycan degradation, and retinol metabolism. Therefore, we speculate that silkworms exhibited both central cell toxicity and stress responses under high concentrations of Chromium exposure. Induction of cytochrome P450 and glutathione metabolism confirmed antioxidant defense participation. At the same time, multiple pathways related to signal transduction, such as the Wnt signaling pathway and ECM receptor interaction, indicated the disruption of ion signaling pathways in normal silkworm cells, possibly affecting the normal functioning of organs. Thus, our findings suggest that Chromium exposure in silkworms elicits a comprehensive stress response that includes metabolism, antioxidative defense, intracellular transport, and cell growth processes.

### 3.4. Key Stress Response and Toxicity Mechanisms

**Figure 5:**
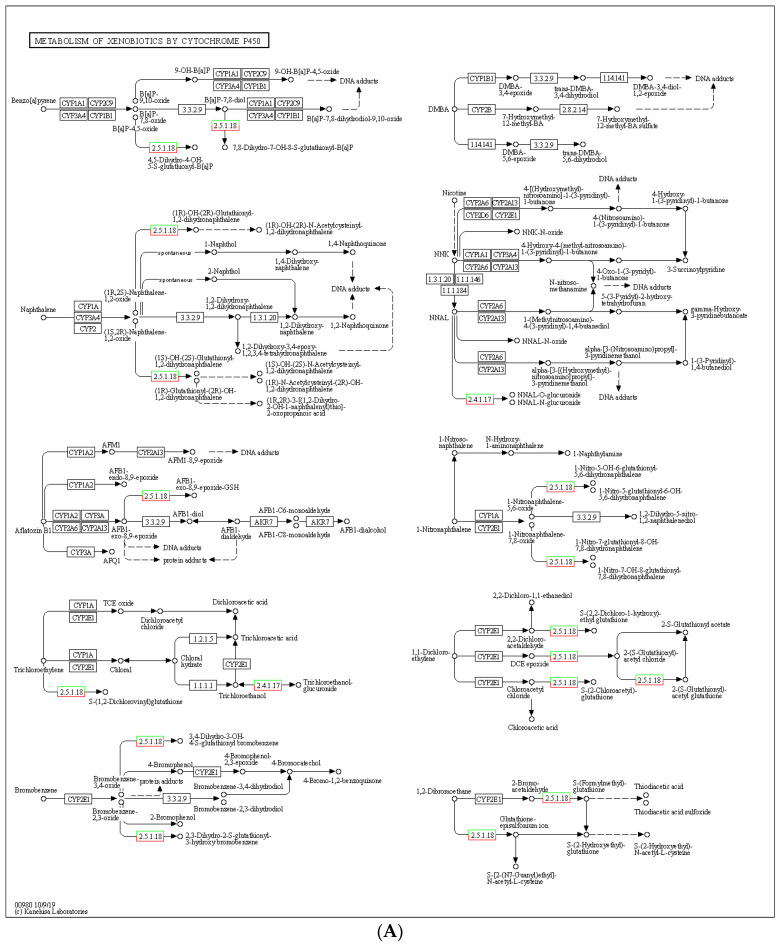

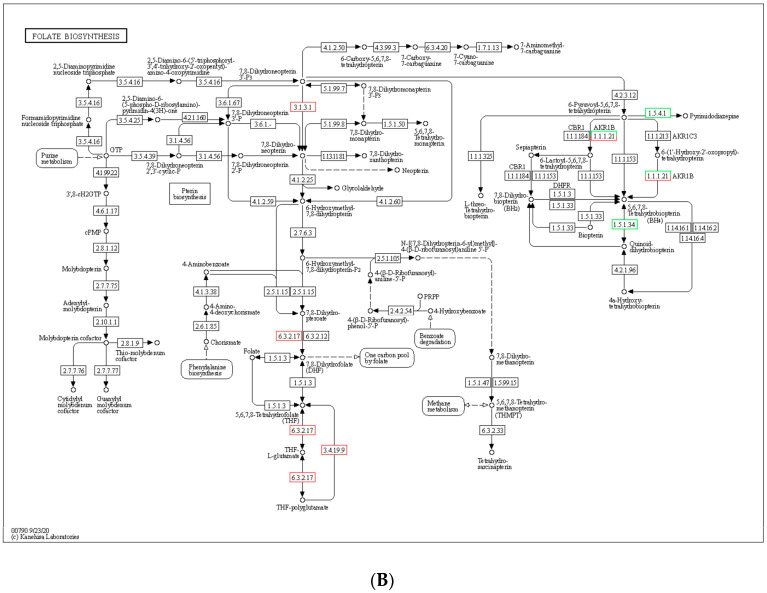
The KEGG map of the metabolism of xenobiotics by cytochrome P450 (**A**) and the peroxisome pathway (**B**). The green boxes represent down-regulated genes, while the red boxes represent up-regulated genes.

To provide more detailed information on specific key terms and genes, we anchored the differentially expressed genes (DEGs) onto KEGG maps (Figure 5A,B) based on the overall mechanisms revealed by KEGG enrichment analysis of medium- (T2B) and high-concentration (T4C) Chromium treatments. We found that the most representative pathway in the T2B group was the metabolism of xenobiotics by cytochrome P450 (map00980) (Figure 4A), which contained genes involved in a variety of antioxidant mechanisms mediated by glutathione compounds and derivatives. The up-regulated and down-regulated genes in this pathway were all distributed in nodes 2.4.1.17 and 2.5.1.18, respectively. Although these genes have similar attributes, our analysis identified specificity in their response to Chromium exposure. Among the specifically activated genes within these nodes, we identified 16 key up-regulated genes, including two types: the UGT (*Ugt2, 33D7, 10288, 013830, 46A2, 40A1, 41A2, 33D2, 10289B, 33D6, UDP-glycosyltransferase precursor, UDP-glucosyltransferase 2-like*), and the GST *(glutathione S-transferase delta 2, GSTe1, GSTd2, GSTs1*). These results suggest that the glucosyltransferase and glutathione S-transferase families are crucial in combating Chromium-induced oxidative stress.

In the T4C group, we investigated the folate biosynthesis pathway (map00790) as an indicator of cellular response to toxicity (Figure 4B). Our analysis revealed that almost all nodes in this pathway underwent gene downregulation, which is a typical sign of biological toxicity. Specifically, we found that nodes 2.8.1.12, 3.4.19.9, 1.1.1.184, 1.1.1.21, and 1.5.4.1 were responsible for the inhibition of biosynthesis of molybdopterin, tetrahydrofolate, tetrahydropterin, tetrahydrobiopterin, and pyrimidodiazepine, respectively. They harmed down-regulated genes, including *GSTo3*, *molybdopterin synthase catalytic subunit isoform X2, carbonyl reductase (NADPH) 3, aldo-keto reductase family one member B1,* and *γ-glutamyl hydrolase A isoform X2*. These results suggest a disruption to critical cellular processes, which could contribute to the toxic effects observed in the T4C group.

### 3.5. Novel Stress Response Genes

In addition to the implications of Chromium exposure on silkworm genes from a comprehensive perspective, we aimed to identify novel genes involved in the stress response that has not been previously characterized or have unknown functional attributes. To do so, we screened for up-regulated genes in the T2B and T4C groups and filtered for those annotated as “uncharacterized” in the genome annotations with only predicted functions in the GO. and KEGG databases. In the T2B group, 18 genes met the selection criteria, and most of them were expected to be indirectly related to stress response, such as genes associated with anatomical structure and epithelial morphogenesis, eye development, and larval or pupal development. However, one gene, *LOC101735895*, is a smell-sensory gene that may directly bind heavy metal ions (Table 2).

In the T4C group, only seven genes met the selection criteria. Apart from the gene *LOC101742664*, associated with proteolysis, the remaining genes are distinct from those in the T2B group. These genes regulate biological and macromolecule metabolic processes, which may aid in the silkworms’ adaptation to stress-induced toxicity. Notably, the *LOC101737268* gene is involved in DNA repair and may be another crucial gene that directly responds to stress (Table 3). This novel gene appears to be activated only under high concentrations of Chromium exposure.

## 4. Discussion

Undertaking a transcriptomic investigation on the effects of Chromium exposure in *B. mori* (silkworm) is of profound significance for several reasons. This research illuminates the molecular intricacies underpinning silkworms’ response and adaptation to heavy-metal stress. Moreover, our findings may inspire the formulation of efficacious breeding strategies that bolster the resilience of *B. mori* and other agriculturally vital insects against heavy metal pollution [17,25,26,37]. In the grander scheme, our study has the potential to extend the current understanding of Chromium toxicity and provide vital insights for preserving the ecological fitness of silkworms and enhancing their survival capabilities in the face of environmental toxicity. We have procured high-quality and dependable data from nine RNA sequencing samples of *B. mori* subjected to two Chromium exposure levels.

Our data exhibit an average sequencing quantity exceeding 6 Gb and a mapping rate surpassing 85%, fulfilling the quality criteria for transcriptome analysis. FPKM distribution and PCA analysis substantiate the superiority of our data, demonstrating comparable FPKM values within each group and the capacity to discern disparities between groups. These outcomes attest to the efficiency and trustworthiness of our experimental design for transcriptome analysis. Additionally, the availability of a reference genome facilitates the direct mapping of sequencing reads to gene models, yielding a more precise quantification of gene expression and minimizing false positives. Consequently, the power of differential gene expression analysis is heightened, allowing for the detection of nuanced yet significant fluctuations in gene expression levels under diverse Chromium concentrations.

Our investigation provides preliminary insights into the influence of Chromium exposure on gene expression in the species *B. mori*. We discovered an intriguing two-phased response under varying levels of Chromium exposure. Under medium Chromium concentrations, an enhanced metabolic response was evident, which was characterized by the amplification of more genes than those undergoing inhibition. In contrast, under high concentrations of Chromium, we observed an emergent survival compromise, documented by a decrease in the number of up-regulated genes, which was outweighed by those undergoing suppression. These observations intimate a hormetic reaction in Bombyx mori upon Chromium exposure, with a toxic damage trajectory plausible between the 50% lethal threshold and the half-lethal dose. Delving further, our Gene Ontology (GO.) enrichment analysis underscored the fundamental role of transporters and transmembrane enzymes in establishing a robust response to stress and adaptive mechanisms. The dichotomy in pathway activation between medium-to-high concentration stress indicates a dose-dependent framework in the stress response mechanism incited by Chromium exposure [38,39]. Insects’ reaction to Chromium exposure could be nonlinear, triggering distinct mechanisms at varying concentrations or governed by a threshold concentration beyond which the system is overwhelmed, thus reducing up-regulated genes and increasing down-regulated genes.

Our investigation delved into the consequential repercussions of chromium on silkworm gene expression profiles, predominantly employing KEGG pathway enrichment analysis of differentially expressed genes (DEGs) under moderate and high chromium concentration scenarios. In silkworms subjected to moderate chromium exposure, gene clusters were notably enriched in pathways linked to pharmaceutical metabolism, carbohydrate metabolism, post-transcriptional modification, antioxidant degradation, and cellular membrane transport. Such enrichment underlines the silkworm’s innate response to manage chromium-induced stress by initiating a variety of metabolic responses and transport methods. Conversely, under the rigors of high chromium concentrations, cellular toxicity and stress responses were dominantly elicited. This was evinced by the conspicuous distribution of DEGs associated with vital developmental pathways such as glycosphingolipid biosynthesis, folate biosynthesis, glycosaminoglycan degradation, and retinol metabolism. Such expression shifts illuminate the potential detrimental implications for the integrity of cellular functions and comprehensive development under elevated chromium exposure. These results lend significant insight into the paradoxical nuance of chromium’s impact, creating a robust foundation for envisaging novel bioremediation strategies.

The observed antioxidant responses in our study are likely a consequence of the cellular stress induced by Chromium exposure. As previously mentioned, Chromium, particularly Cr(VI), generates ROS within the cell, leading to oxidative stress. This oxidative stress activates the cytochrome P450 and the peroxisome pathway, which triggers the transcriptional up-regulation of antioxidant genes. The increased expression of antioxidant enzymes, such as SOD, GST, and catalase, helps to scavenge ROS and protect cellular macromolecules from oxidation [1,11,12,40]. In our investigation, we elucidated the engagement of antioxidant defense and multiple signaling pathways, underscoring the nuanced and sophisticated nature of silkworms’ physiological stress response mechanisms when confronted with Chromium exposure. These intricate physiological responses draw attention to the significant biological pressures endured by silkworms in Chromium-rich environments, potentially having deleterious impacts on their growth and development dynamics. Presented against a backdrop of preceding studies focusing on heavy metal effects, our revelations provide fresh, compelling insights, proficiently extending and enriching earlier associations and conclusions [21,41].

The analysis of differentially expressed genes under varied Chromium exposure concentrations unraveled distinct biological pathways and genes involved in silkworms’ physiological responses to such stress. Notably, our KEGG pathway analysis indicated that xenobiotics metabolism by cytochrome P450 was the most representative pathway in the medium concentrate treatment group. Within this pathway, up-regulated and down-regulated genes in nodes 2.4.1.17 and 2.5.1.18 are vital for combating Chromium-induced oxidative stress. We identified 16 key up-regulated genes, including glucosyltransferase and glutathione S-transferase families, playing pivotal roles in antioxidative mechanisms. Under previous studies, the up-regulation of genes in these enzyme families responding to Chromium exposure suggests their importance in cellular responses to oxidative stress and detoxification [28,42]. Glucosyltransferase enzymes aid the detoxification and elimination of xenobiotics by glycosylating substrates, while glutathione S-transferase enzymes catalyze reduced glutathione conjugation to oxidized substrates, shielding cells from oxidative damage. Their activation in response to Chromium exposure indicates potential as future biomarkers for environmental contamination monitoring.

Conversely, the high concentrate group exhibited significant down-regulation in the folate biosynthesis pathway, indicating biological toxicity. The down-regulated vital nodes in this pathway reveal disruptions in critical cellular processes, potentially contributing to the observed toxic effects. The folate biosynthesis pathway’s down-regulation concerning Chromium exposure signifies its role in cellular toxicity response [43,44,45]. Specifically, the inhibited biosynthesis of molybdopterin, tetrahydrofolate, tetrahydropterin, tetrahydrobiopterin, and pyrimidodiazepine within key nodes suggests disruptions in vital cellular processes, which may account for the toxicity observed in the high-concentrate group.

The discovery of novel genes implicated in stress response is vital for understanding the mechanisms underlying organisms’ adaptation and responses to environmental stressors like Chromium contamination [25,28,42,46]. Recognizing new genes could reveal previously unknown pathways and mechanisms to manage stress-induced toxicity. Moreover, novel gene discoveries may lead to the development of new biomarkers for monitoring environmental contamination or as targets for creating innovative bioremediation strategies.

The disparity in the number and types of novel genes identified between the medium and high concentrate groups might result from the varying Chromium exposure levels the silkworms faced. With higher concentrations in the high-concentration group, the silkworms’ response may have been more severe and potentially more pronounced. The upregulation of genes linked to DNA repair and proteolysis in the high-concentrate group suggests a more direct approach to mitigating toxicity at elevated stress levels. In contrast, the medium concentrate group’s up-regulated genes, associated with the anatomical structure or metabolic processes, may indicate a subtler response to lower stress levels.

Nonetheless, further studies are crucial for comprehending the differences between the groups and the relevance of the novel genes identified in each group. These findings contribute a fresh perspective to the study of Chromium exposure in silkworms, shedding light on their unique genetic response mechanisms.

## 5. Conclusions

This study investigated the effects of Chromium exposure on *B. mori* (silkworm) via transcriptomic analysis of nine samples in different treatment groups. The data, derived from nine RNA sequencing samples, revealed an adaptive response to low-dose Chromium exposure and a toxicity-damaged mode at higher concentrations. GO enrichment analysis demonstrated the involvement of transport, transmembrane-related enzymes, and stress response mechanisms. In contrast, KEGG pathway analysis showed a dose-dependent response activating metabolic and transport processes at medium Chromium levels and cell toxicity and stress response mechanisms at higher levels. Notably, up-regulated genes in the xenobiotics metabolism pathway by cytochrome P450 and antioxidant enzymes, glucosyltransferase, and glutathione S-transferase helped combat Chromium-induced oxidative stress. In contrast, high Chromium levels led to the down-regulation of the folate biosynthesis pathway, indicating biological toxicity. Two novel genes *LOC101742664* and *LOC101737268* were also recognized, which could facilitate future stress-adaptation understanding and biotoxicity effect evaluations in silkworms.

## Figures and Tables

**Table 1:** The sequencing and mapping statistics for nine retrieved RNA-seq samples.

Sample	Number of Clean Reads	Clean Data (bp)	Clean Reads (%)	Mapped Reads	Mapping Rate (%)
TKAa1	46,480,154	7,018,503,254	94.1	44,133,117	94.95
TKAb2	49,383,830	7,456,958,330	94.63	47,462,432	96.11
TKAc3	46,228,046	6,980,434,946	94.86	44,424,220	96.1
T2Ba1	44,269,598	6,684,709,298	93.73	39,665,480	89.6
T2Bb2	34,848,742	5,262,160,042	94.84	31,339,902	89.93
T2Bc3	43,443,810	6,560,015,310	94.24	39,661,947	91.29
T4Ca1	40,528,222	6,119,761,522	94.93	39,290,214	96.95
T4Cb2	38,550,946	5,821,192,846	94.84	37,167,990	96.41
T4Cc3	38,875,430	5,870,189,930	94.91	37,722,658	97.03

Note: The a1, b2, and c3 followed after the sample names represent the three sample replicates. The mapping rate was calculated using the number of mapped reads against the total clean reads.

**Table 2:** Novel stress response genes in the T2B group.

Gene ID	B.P. Terms
LOC101744812	GO:0006629//lipid metabolic process; GO:0006638//neutral lipid metabolic process
LOC101747119	GO:0009653//anatomical structure morphogenesis
LOC119628334	GO:0001654//eye development; GO:0007166//cell surface receptor signaling pathway; GO:0007185//transmembrane receptor protein tyrosine phosphatase signaling pathway
LOC101740376	GO:0007268//chemical synaptic transmission; GO:0007270//neuron-neuron synaptic transmission
LOC101735576	GO:0002009//morphogenesis of an epithelium; GO:0002165//instar larval or pupal development
LOC101742664	GO:0006508//proteolysis; GO:0006511//ubiquitin-dependent protein catabolic process; GO:0006807//nitrogen compound metabolic process
LOC101740250	GO:0001654//eye development; GO:0001745//compound eye morphogenesis; GO:1903430//negative regulation of cell maturation; GO:2000242//negative regulation of reproductive process
LOC101742357	GO:0000003//reproduction; GO:0002064//epithelial cell development
LOC101746579	GO:0000003//reproduction; GO:0000278//mitotic cell cycle
LOC101739530	GO:0000086//G2/M transition of mitotic cell cycle; GO:0007346//regulation of mitotic cell cycle
LOC105841728	GO:0001736//establishment of planar polarity; GO:0001737//establishment of imaginal disc-derived wing hair orientation; GO:0001738//morphogenesis of a polarized epithelium
LOC119628328	GO:0007155//cell adhesion; GO:0007157//heterophilic cell–cell adhesion via plasma membrane cell adhesion molecules
LOC101736099	GO:0007346//regulation of mitotic cell cycle; GO:1902749//regulation of cell cycle G2/M phase transition; GO:1902750//negative regulation of cell cycle G2/M phase transition;
LOC101742038	GO:0001703//gastrulation with mouth forming first
LOC101739155	GO:0000462//maturation of SSU-rRNA from tricistronic rRNA transcript (SSU-rRNA, 5.8S rRNA, LSU-rRNA)
LOC101735895	GO:0007600//sensory perception; GO:0007606//sensory perception of chemical stimulus; GO:0007608//sensory perception of smell
LOC101742065	GO:0002009//morphogenesis of an epithelium; GO:0002165//instar larval or pupal development
LOC101740587	GO:0000375//RNA splicing, via transesterification reactions

Note: The primary GO. accessions and the corresponding functional descriptions are shown.

**Table 3:** Novel stress response genes in the T4C group.

Gene ID	B.P. Terms
LOC101737268	GO:0006281//DNA repair; GO:0006950//response to stress; GO:0006974//cellular response to DNA damage stimulus
LOC101746842	GO:0006491//N-glycan processing; GO:0006807//nitrogen compound metabolic process
LOC101742664	GO:0006508//proteolysis; GO:0006511//ubiquitin-dependent protein catabolic process; GO:0006807//nitrogen compound metabolic process
LOC119631147	GO:0050789//regulation of biological process; GO:0060255//regulation of macromolecule metabolic process
LOC101735873	GO:0006935//chemotaxis; GO:0007155//cell adhesion; GO:0007157//heterophilic cell–cell adhesion via plasma membrane cell adhesion molecules
LOC101746799	GO:0006810//transport; GO:0006897//endocytosis; GO:0006909//phagocytosis
LOC101742993	GO:0007043//cell–cell junction assembly; GO:0007275//multicellular organism development; GO:0007424//open tracheal system development

Note: The primary GO. accessions and the corresponding functional descriptions are shown.

## Data Availability

All raw data was uploaded to the China National Center for Bioinformation Genome Sequence Archive database under the Bioproject accession of PRJCA018238.

## Supplementary Materials

The following supporting information can be downloaded at: https://www.mdpi.com/article/10.3390/genes14081616/s1, Table S1: The DEGs of the T2B group against the TKA group; Table S2: The DEGs of the T4C group against the TKA group; Table S3: The functional gene annotations of the reference genome; Table S4: G.O. enrichment for the DEGs of the T2B group against the TKA group; Table S5: KEGG enrichment for the DEGs of the T2B group against the TKA group; Table S6: G.O. enrichment for the DEGs of the T4C group against the TKA group; Table S7: KEGG enrichment for the DEGs of the T2B group against the TKA group.
